# Fano resonance of Li-doped KTa_1−*x*_Nb_*x*_O_3_ single crystals studied by Raman scattering

**DOI:** 10.1038/srep23898

**Published:** 2016-04-06

**Authors:** M. M. Rahaman, T. Imai, T. Sakamoto, S. Tsukada, S. Kojima

**Affiliations:** 1 Graduate School of Pure and Applied Sciences, University of Tsukuba, Tsukuba, Ibaraki 305-8573, Japan; 2 NTT Corporation Device Innovation Center, Nippon Telegraph and Telephone Corporation, Atsugi, Kanagawa 243-0198, Japan; 3 Faculty of Education, Shimane University, Matsue, Shimane 690-8504, Japan

## Abstract

The enhancement of functionality of perovskite ferroelectrics by local structure is one of current interests. By the Li-doping to KTa_1−*x*_Nb_*x*_O_3_ (KTN), the large piezoelectric and electro-optic effects were reported. In order to give new insights into the mechanism of doping, the microscopic origin of the Fano resonance induced by the local structure was investigated in 5%Li-doped KTN single crystals by Raman scattering. The coupling between the continuum states and the transverse optical phonon near 196 cm^−1^ (Slater mode) caused a Fano resonance. In the vicinity of the cubic-tetragonal phase transition temperature, *T*_C-T_ = 31 °C, the almost disappearance of the Fano resonance and the remarkable change of the central peak (CP) intensity were observed upon heating. The local symmetry of the polar nanoregions (PNRs), which was responsible for the symmetry breaking in the cubic phase, was determined to *E*(*x*, *y*) symmetry by the angular dependence of Raman scattering. The electric field induced the significant change in the intensity of both CP and Fano resonance. From these experimental results, it is concluded that the origin of the Fano resonance in Li-doped KTN crystals is the coupling between polarization fluctuations of PNRs and the Slater mode, both belong to the *E*(*x*, *y*) symmetry.

The lead-based ferroelectrics have been extensively used in our daily lives for their colossal piezoelectricity and giant dielectric response; however, its future applicability is highly limited because of the toxic nature of lead. Therefore, the advancements of lead free ferroelectrics materials are an imperative matter in applied physics. In order to enhance the functionality of lead free ferroelectrics, the role of local structure is very important^1^. In the midst of lead free ferroelectrics, the investigation of Li-doped KTa_1−*x*_Nb_*x*_O_3_ (KTN) has fascinated much scientific attention owing to their enormous quadratic electro-optic coefficient^2^,^3^,^4^, good photorefractive effect^5^, and excellent piezoelectric effect^6^, which make them one of the potential candidates for not only optical but also electromechanical device applications.

KTN is the solid solution of KTaO_3_ and KNbO_3_. The most important aspect of KTN is the off-center displacements of Nb ions at the B-site and therefore they induce polar naoregions (PNRs)^7^. The physical origin of the off-center displacements of Nb ions was rationalized by the pseudo Jahn-Teller effect (PJTE)^8^. A light scattering study also explained the off-center displacements of Nb ions in KTN by eight site model, in which Nb ions displace among the equivalent [111] directions^9^. As widely reported by various kinds of measurements^10^,^11^,^12^,^13^,^14^,^15^,^16^,^17^, one off-center displacement interacts with the neighboring off-center displacements, leading to the local polar structure called PNRs in the paraelectric cubic phase of KTN crystals. The PNRs are characterized by various temperatures in the cubic phase. First, the Burns temperature, *T*_B_^18^, below which the dynamic PNRs appear. Second, the intermediate temperature, T*, at which the dynamic PNRs start to transform into static PNRs^10^,^11^,^12^.

Recently, the effect of Li-doping in KTaO_3_ and KNbO_3_ is one of the interesting research fields in materials science. In K_1-*y*_Li_*y*_TaO_3_ (KLT) and K_1-*z*_Li_*z*_NbO_3_ (KLN), the substitutional Li ions occupy one of six off-center sites along the [100] directions at the A-site^19^,^20^, which can lead to the formation of random fields that enhance the appearance of PNRs. In KLT, there is a crossover at around critical concentration *y* = 0.022 between a freezing and a structural transition with a critical level of local polarization^19^. Most recently, Rahaman *et al.* observed the effects of Li-doping on elastic properties, which enhanced the relaxor nature by the growth of PNRs of KTN^21^. Therefore, the Li-doped KTN is an intriguing topic to investigate the Li-doping effects on precursor dynamical properties of a relaxor ferroelectric phase transition.

In KTN, the dynamical aspect of PNRs was extensively studied by inelastic light scattering^10^,^11^,^13^,^14^,^22^. However, the microscopic origin of the Fano resonance at around 196 cm^−1^ in Li-doped KTN still remains fuzzy. The phenomenon of a Fano resonance results from the interaction between a discrete state and continuum states showing the asymmetry of the spectral line shape^23^. The possible continuum states, which can interfere with the optical phonons and give rise to a Fano resonance, are the two acoustical phonon state^24^, another transverse optic (TO) phonon via acoustic phonons^25^, and the rapid polarization fluctuations with the frequency up to the Fano resonance frequency within micro polar regions^26^, and nanoscopic polar regions^27^. The Fano resonance was also observed in different types of materials from several physical origins by the various measurements^28^,^29^,^30^. In this study, we investigated the microscopic origin of the Fano resonance in Li-doped KTN crystals using the temperature, angular, and electric field dependences of Raman scattering. The local symmetry breaking in the cubic phase was discussed on the basis of the existence of the first order Raman modes connecting with the evolution of the PNRs.

## Methods

The K_0.95_Li_0.05_Ta_0.73_Nb_0.27_O_3_ (KLTN/0.05/0.27) and K_0.95_Li_0.05_Ta_0.74_Nb_0.26_O_3_ (KLTN/0.05/0.26) single crystals used in the present study were grown by the top seeded solution growth technique at NTT Corporation. The crystals were cut having the size of 4.0 × 3.2 × 1.2 mm^3^ and 4.0 × 3.18 × 1.0 mm^3^ with the largest faces perpendicular to the [100] direction, respectively. In KLTN/0.05/0.26 crystal, the platinum electrodes were deposited on the faces perpendicular to the [100] direction to apply the electric field. The temperature and electric field dependences of Raman spectra were measured using a double monochromator (Horiba-JY, U-1000) with the resolution of 1 cm^−1^ at a back scattering geometry. The Porto’s notation like *ā*(*cc*)*a* (VV) and *ā*(*bc*)*a* (VH) is used to denote the scattering configurations, where *ā* and *a* represent the direction of propagation of incident and scattered light, respectively. In the case of VV scattering the directions of the polarization of incident and scattered light are parallel to each other represented by, for example, *cc*. However, in the case of VH scattering the directions of the polarization of incident and scattered light are perpendicular to each other, for example, along *c* and *b* axes, respectively, represented by *bc*. A diode pumped solid state (DPSS) laser with a wavelength of 532 nm was used to excite the samples. The temperature of the samples was controlled by the heating/cooling stage (Linkam, THMS600) with ±0.1 °C accuracy over all temperatures.

**Figure 1:**
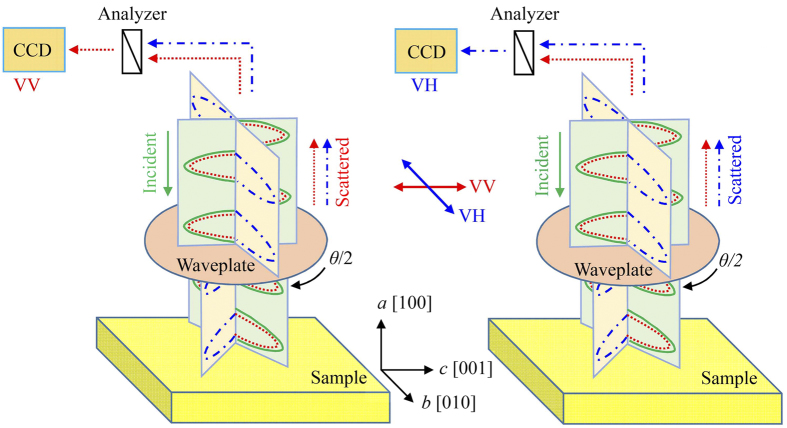
A typical sketch of the experimental scattering geometry with a half-wave plate. The orthogonal coordinates (**a–c**) represent the experimental coordinates, where the incident light propagating along *ā* had a polarization direction along c axis. When the polarization direction of incident light is inclined *θ*/2 with respect to the optical axis of the waveplate, the polarization plane of incident is rotated by *θ* while that of the scattered light rotates by −*θ*.

For the angular dependence of Raman scattering, the KLTN/0.05/0.27 crystal was put on the *xyz* mapping stage (Tokyo Instruments) inside the Linkam installed in the optical reflection microscope (Olympus). The linearly polarized light from DPSS was incident to the sample through a polarization rotation device (Sigma Koki) equipped with a broadband half-waveplate (Kogakugiken). When the polarization direction of incident light is inclined *θ*/2 with respect to the optical axis of the waveplate, the waveplate rotates polarization plane of incidence *θ* degree. On the other hand, the polarization direction of scattering light, propagating in opposite direction of incident light, is rotated by −*θ* degree. For better understanding the configurations of scattering, a typical illustration of the experimental scattering geometry is shown in Fig. 1. The strong elastic scattering was highly reduced by two volume Bragg gratings so called “ultra narrow-band notch filters” (OptiGrate). The inelastic scattering light was dispersed by a single-monochromator (Lucir) and the dispersed component was detected using a charge coupled device (CCD, Andor).

## Results

### Temperature dependence of Raman scattering spectra

**Figure 2:**
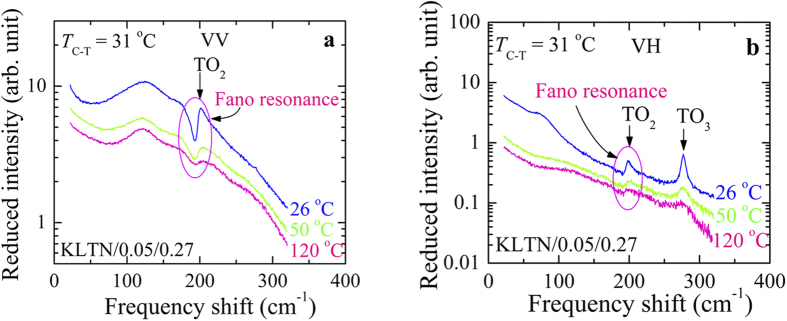
Reduced (**a**) *ā*(*cc*)*a* (VV) and (**b**) *ā*(*bc*)*a* (VH) Raman scattering spectra at some selected temperatures of the KLTN/0.05/0.27 single crystal.

In the analysis of Raman scattering spectra, the reduced intensity, *I*^r^(*ω*), was calculated from the Stokes component of Raman scattering intensity by the following equation.


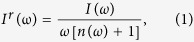


where 
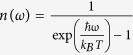
 is the Bose-Einstein population factor, in which *k*_B_ is the Boltzmann constant and *ħ* is the Dirac constant. *I*(*ω*) is the observed Raman scattering intensity. By the reduced intensity, the effect of phonon population on spectra can be avoided. The temperature dependence of reduced *ā*(*cc*)*a* (VV) and *ā*(*bc*)*a* (VH) Raman spectra of a Li-doped KTN i.e., K_0.95_Li_0.05_Ta_0.73_Nb_0.27_O_3_ (KLTN/0.05/0.27) single crystal is shown in Fig. 2. The existence of a prominent first order TO_2_ mode at about 196 cm^−1^ in both VV and VH spectra and a TO_3_ mode at around 277 cm^−1^ at 26 °C demonstrates that the symmetry of the KLTN/0.05/0.27 single crystal is not cubic *Pm-3m* symmetry at room temperature (RT). The detailed mode assignment of the KTN single crystals were studied in Refs. 31, 32, 33, 34. According to the phase diagram of KTN^35^,^36^, a cubic phase is indeed expected for the non-doped KTN/0.27 crystal at RT. However, Li-doping raises the cubic-tetragonal phase transition temperature (*T*_C-T_) and the tetragonal phase with *P4mm* symmetry was reported at RT in KLTN/0.05/0.27^21^. By Raman scattering, the effect of Li-doping on the phase transition of the KTN crystals was also reported by Prater *et al.*^37^.

**Figure 3:**
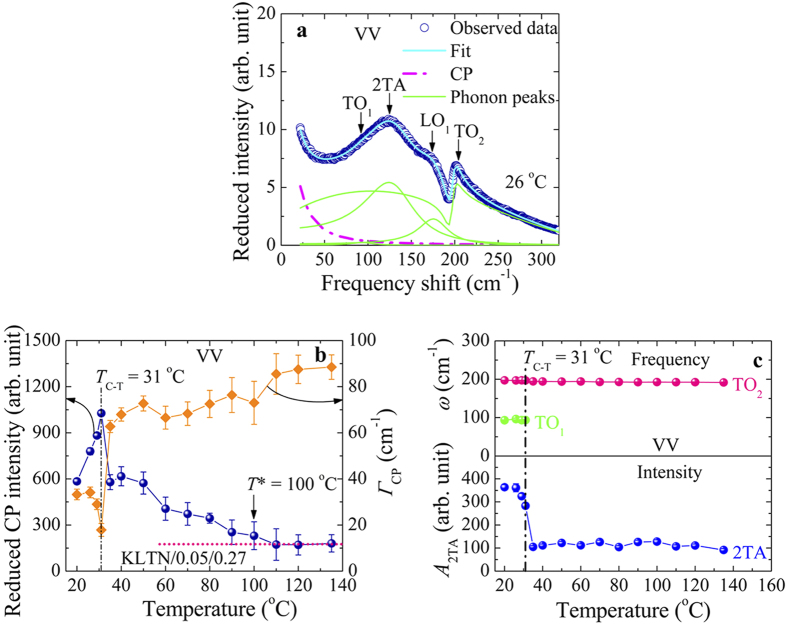
(**a**) The example of a fit of Raman spectrum using the equation (2). (**b**) Temperature dependence of the integrated reduced intensity and FWHM of the CP of the KLTN/0.05/0.27 single crystal at the VV scattering. The dotted and dash-dotted lines in (**b**) are guide to eyes. (**c**) The frequency shift of the first order TO_1_ and TO_2_ modes (upper half) and the variation of the intensity of the 2TA mode (lower half) at the VV scattering of the KLTN/0.05/0.27 crystal as a function of temperature.

To investigate the temperature dependence of a central peak (CP), which is related to the relaxation process of dynamic PNRs, all spectra were fitted by the combination of a Lorentzian CP, damped harmonic oscillator (DHO) model, and a third order polynomial with the Fano function as follows^22^,^38^


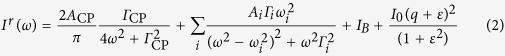


where *I*_B_ = *P*(*ω* − *ω*_TO2_)^3^ + *Q*(*ω* − *ω*_TO2_)^2^ + *R*(*ω* − *ω*_TO2_) + *S*, *A*_CP_ and *Γ*_CP_ are intensity and FWHM (full width at half maximum) of the CP, respectively. The *A*_i_, *Г*_i_, and *ω*_i_ are intensity, damping, and frequency of the *i*th Raman mode, respectively. The *I*_0_ is intensity of the Fano resonance (TO_2_ mode), *q* is the asymmetry parameter characterises the coupling strength between the phonon and continuum states, *ε* = 2(*ω* − *ω*_TO2_)/*Γ*_TO2_ is the reduced energy, where *Γ*_TO2_ is the FWHM of the Fano resonance. *P*, *Q*, *R*, and *S* are the constants. Figure 3a shows the fit result using equation (2). Since the Raman modes were best resolved at the lowest temperature, therefore, the fitting procedure was started with the spectrum measured at the lowest temperature. The best fit in the preceding temperature was taken as the initial data for the next higher temperature. The temperature dependence of the reduced intensity, *A*_CP_, and FWHM*, Г*_CP_, of the CP observed in the VV scattering spectrum is shown in Fig. 3b. As can be seen in Fig. 3b, the *A*_CP_ begins to increase below the intermediate temperature, T* ∼ 100 °C, upon cooling, reflecting the sudden growth of the volume fraction of PNRs^10^,^11^,^21^. In Fig. 3b, the temperature dependence of *Г*_CP_, which is inversely proportional to the relaxation time, becomes narrower remarkably towards the *T*_C-T_ upon cooling, implying the slowing down of the relevant polarization fluctuations of PNRs. Therefore, the anomaly at around 100 °C must reflect a significant change in the dynamical properties of the KLTN/0.05/0.27 crystal upon cooling. It is significant that both *A*_CP_ and *Г*_CP_ exhibit clear anomaly in the vicinity of the *T*_C-T_ = 31 °C. Moreover, the first order TO_1_ mode in the VV spectra completely vanished at *T*_C-T_ upon heating (Fig. 3c), which can be the clear indication of the phase transition of the KLTN/0.05/0.27 single crystal. The similar variation of the intensity and the *Г*_CP_ of the CP associated with the precursor dynamics was also observed in Pb(Zn_1/3_Nb_2/3_)O_3_ (PZN) single crystal^38^. The values of the characteristic temperatures *T*_C-T_ and T* are in good agreement with the values reported in ref. 21.

**Figure 4:**
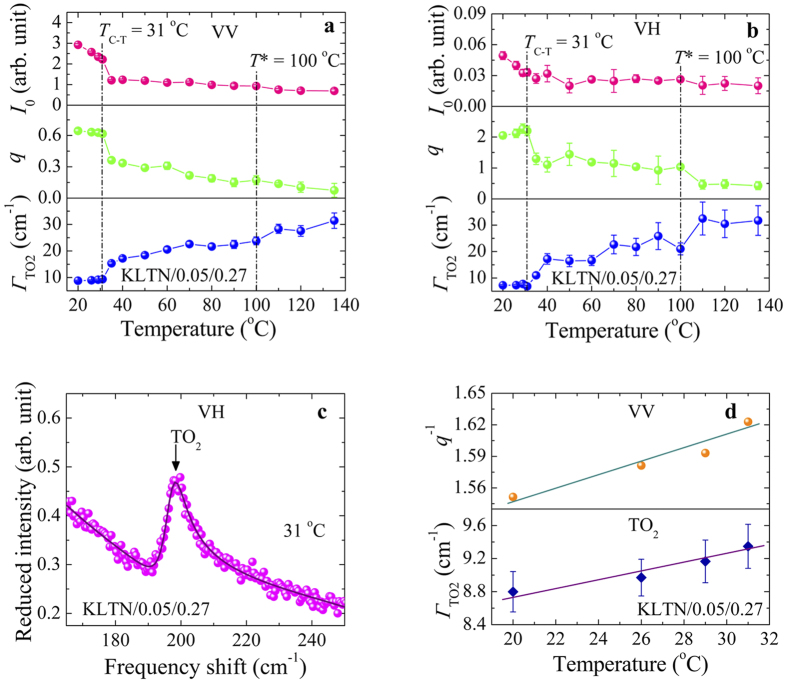
The temperature dependence of reduced intensity, *I*_0_, line shape parameter, *q*, and FWHM, *Г*_TO2_ of the Fano resonance in (**a**) VV and (**b**) VH scattering spectra of the KLTN/0.05/0.27 crystal fitted by equation (2). The example of a fitted curve is shown in (**c**). (**d**) The temperature dependence of *q*^−1^ and *Γ*_TO2_ observed at the VV scattering geometry in the ferroelectric phase. The solid lines in (**d**) are guide to eyes.

**Figure 5:**
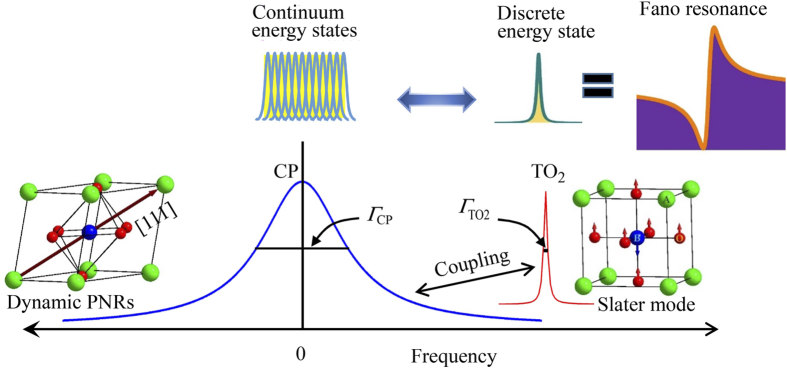
A schematic illustration of the coupling between slow-relaxing CP (a broad spectrum indicates continuum states) and TO_2_ phonon (a sharp spectrum indicates discrete state). The *Γ*_CP_ and *Γ*_TO2_ are the relaxation rates of the CP and TO_2_ phonon, respectively, (*Γ*_CP_ > *Γ*_TO2_). The displacement pattern of Slater mode is depicted in the right side, whereas dynamic PNRs is displayed in the left side. The local polarization of PNRs fluctuates along the equivalent [111] directions indicated by an arrow.

It is worth noting that the temperature dependence of the reduced intensity, *I*_0_, of the Fano resonance decreases rather fast in the vicinity of the *T*_C-T_ upon heating, while it is still intense above the *T*_C-T_ (Fig. 4a,b), indicating the symmetry breaking caused by the dynamic PNRs. In a typical relaxor, the breaking of symmetry in the cubic phase due to the existence PNRs with rhombohedral *R3m* symmetry was also studied by Raman scattering^39^,^40^. It is also significant that the *Г*_TO2_ shows the noticeable change associated with these precursor effects, therefore, the Fano resonance might be correlated with the PNRs. Since the polarization fluctuations of the PNRs give rise to the CP, hence, the coupling between the CP and the TO_2_ phonon can cause a Fano resonance near 196 cm^−1^ in Li-doped KTN crystals. The schematic illustration of the coupling phenomenon between CP and TO_2_ phonon is shown in Fig. 5.

### Angular dependence of Raman scattering spectra

**Figure 6:**
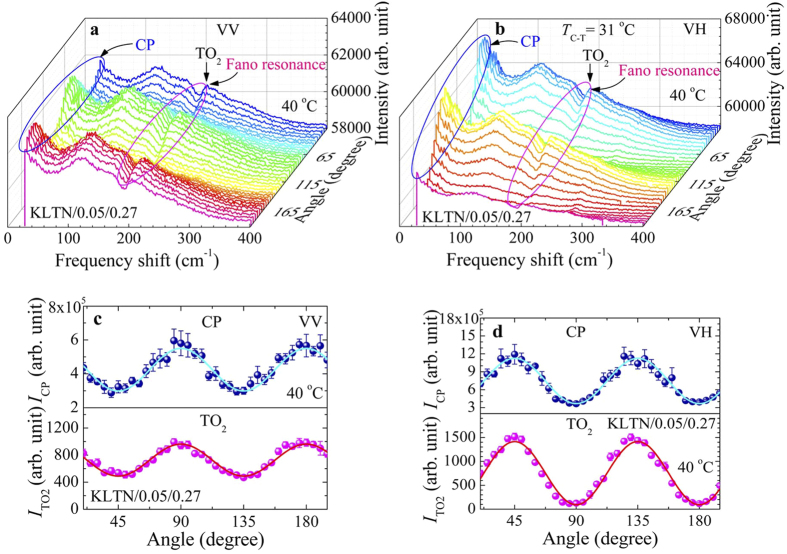
Angular dependence of (**a**) VV and (**b**) VH Raman scattering spectra of the KLTN/0.05/0.27 single crystal measured at 40 °C. The observed intensity of the CP (upper half) and the Fano resonance (lower half) in (**c**) VV and (**d**) VH spectra as a function of rotation angle. The solid lines in (**c**) and (**d**) are the best fitted curves using the equations (5) and (6), respectively.

For better understanding the physical origin of the Fano resonance in Li-doped KTN crystals, we analyzed the angular dependence of both VV and VH spectra in the cubic phase as shown in Fig. 6a,b, respectively. The angular dependence of Raman spectra shows the periodic variation with the rotation of the plane of polarization, and the variation of the intensity was out of phase between VV and VH spectra. We are mainly concerned about the microscopic origin of the Fano resonance. The angular dependence of Raman spectra was analyzed on the basis of Raman tensor calculations assuming the local symmetry of PNRs to clarify the physical origin of the CP and the Fano resonance in the cubic phase. According to the neutron pair distribution function analysis, the local symmetry of the PNRs of the Pb(Mg_1/3_Nb_2/3_)O_3_ (PMN) crystal was reported as rhombohedral *R3m*^41^. The *R3m* symmetry has three Raman active modes, *A*_1_(*z*), *E*(*y*), and *E*(*-x*) with the following Raman tensors^42^:


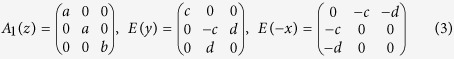


The angular dependence of Raman integrated intensity was fitted by the following expression


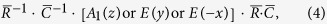



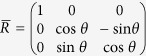


where *θ* is the rotation angle of the experimental coordinates and 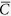 is the transformation matrix for the modification of the Raman tensor components^42^. Since the local rhombohedral regions are oriented randomly along the eight equivalent polarization directions, thus the angular dependence was calculated in the multi-domain states in which the contributions of all eight domains are summed up equaly^42^. The angular dependence of observed Raman intensity of the CP, *I*_CP_, and the Fano resonance, *I*_TO2_, in both VV and VH spectra in the cubic phase are plotted in Fig. 6c,d, respectively. It is worth noting that the change of the intensity of the CP and the Fano resonance with the rotation of the plane of polarization in the cubic phase of the Li-doped KTN crystal is similar to the behaviour of PNRs of PMN with the *E*(*x, y*) symmetry^42^. Hence, on the analysis of the angular dependence of Raman results of the Li-doped KTN crystals, we excluded the *A*_1_(*z*) symmetry of PNRs. For the *E*(*x, y*) symmetry of PNRs, one can obtain the values of the intensity of the *cc* (VV) and *bc* (VH) components after the transformation of the Raman tensor component using the equation (4) given by


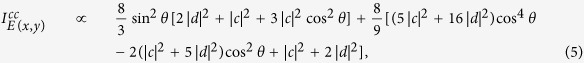



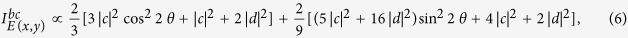


The change of the intensity of the CP and the Fano resonance observed at both VV and VH spectra is well fitted by the theory via equations (5) and (6) as shown in Fig. 6c,d, respectively. The fitted curves reproduce the observed intensity variations rather well, implying that the CP and the Fano resonance in the cubic phase stem from the *E*(*x, y*) symmetry of PNRs with a rhombohedral *R3m* symmetry. Since both the CP and the TO_2_ phonon belong to the same symmetry, therefore, the CP and the TO_2_ phonon can be coupled with each other resulting in a Fano resonance in Li-doped KTN single crystal.

### Electric field induced Raman scattering spectra

**Figure 7:**
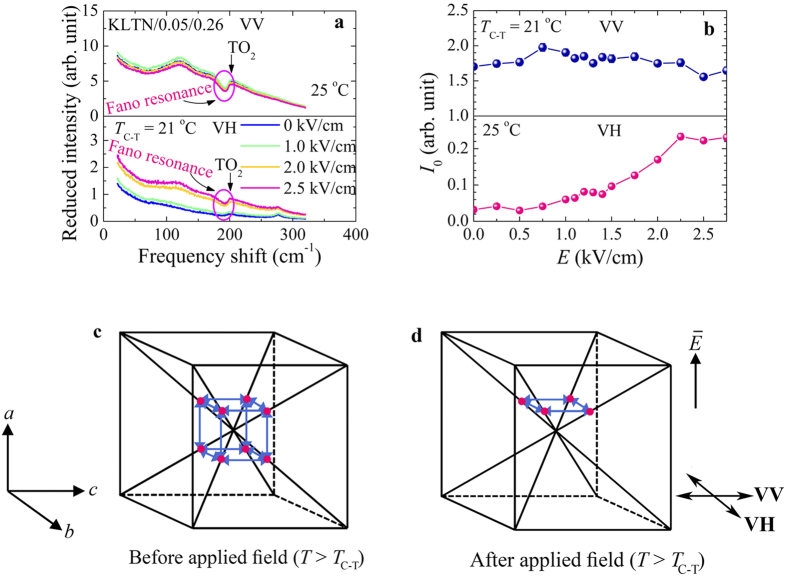
(**a**) Electric field dependence of both VV (upper half) and VH (lower half) Raman spectra of the KLTN/0.05/0.26 single crystal measured at 25 °C. (**b**) The reduced intensity of the Fano resonance as a function of the electric field in both VV (upper half) and VH (lower half) spectra measured at 25 °C. A schematic illustration of off-center displacements of Nb ions in the cubic phase of the Li-doped KTN crystals in (**c**) before applied field and (**d**) after applied field. The off-centering of Li ions among the equivalent symmetry related sites, albeit not shown in the figure, can be described in a similar way.

In order to clarify the origin of the Fano resonance, the electric field dependence of Raman scattering spectra of the KLTN/0.05/0.26 single crystal was investigated above and below the *T*_C-T_ = 21 °C. The lower value of the *T*_C-T_ of the KLTN/0.05/0.26 crystal than that of the KLTN/0.05/0.27 crystal occurs by the lower Nb concentrations. Figure 7a shows the electric field dependence of Raman spectra of the KLTN/0.05/0.26 crystal in both VV and VH scattering measured at 25 °C. It is interesting to note that the application of the electric fields causes the increase of the intensity for the VH spectra in the cubic phase, while no noticeable change appears for the VV spectra. In KTN, the relaxor like behaviour is originated from the correlated motion of the off-center Nb ions. These displacements are along equivalent [111] directions, and each one can switch between several equivalents or symmetry related sites as shown in Fig. 7c^7^,^13^. The motion between these various orientations affects the off-diagonal components of the polarizability tensors, and therefore influences the intensity of the VH scattering. In this study, the electric field was applied along the *a* i.e., [100] direction, which is orthogonal to the *b*-*c* scattering plane. As a result, Nb ions became constrained for moving in this plane i.e., amongst four equivalent positions (Fig. 7d). Such a motion enhances the intensity of the VH scattering, while preventing that of the VV one. This effect is clearly seen in Fig. 7b, where the *I*_0_ increases with increasing electric field in the VH spectrum, while in the VV spectrum the *I*_0_ does not exhibit any appreciable change by the electric field. The switching of the Nb ions amongst four equivalent sites under the moderate electric fields can be the evidence of the dynamical response of local polarizations in the PNRs. Recently, the dynamic response of the PNRs under an electric field in the cubic phase of KTN was reported in Ref. 43. It is also expected that the restriction of the Nb ions to four sites results in a [100] time-averaged polarizations with the application of the sufficient applied field, and resulting in a crystal transforms into a lower tetragonal *P4mm* symmetry. Since the applied electric filed was not as high as that can induce the phase transition of the KLTN/0.05/0.26 crystal at 25 °C, therefore the Raman spectra below 2.75 kV/cm was not same as observed spectra in the tetragonal phase, as presented in Fig. 2.

**Figure 8:**
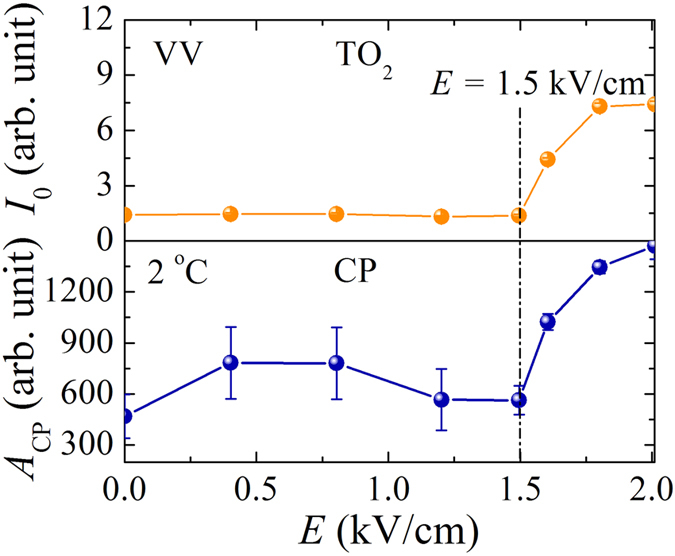
The electric field dependence of the reduced intensity of the Fano resonance (upper half) and the CP (lower half) measured at 2 °C.

To clarify the effects of the electric field on the Fano resonance of the Li-doped KTN crystals, we also performed the field induced Raman scattering in the ferroelectric phase where the polarization fluctuations of PNRs were frozen into nano-domain states, while the growth into macrodomains was blocked by the random fields. The electric field dependence of the *I*_0_ and the *A*_CP_ measured at 2 °C is displayed in Fig. 8. It is important to note that the *I*_0_ and the *A*_CP_ exhibit the anomalous change at *E* = 1.5 kV/cm. In Li-doped KTN, without the external electric field there was no driving force for switching the nano-domain states to ferroelectric macro-domains in the tetragonal phase. Since the applied electric field along [100] direction stabilizes a tetragonal phase, therefore, the electric filed induced sudden increase of the *I*_0_ and the *A*_CP_ is caused by the alignment of the random nano-domain states along the field direction. Hence, the field induced change at 1.5 kV/cm can be the transition from random nano-domain states to a macro-domain state. By the applied electric field, such a transition from nano-domain states to a macro-domain state was also observed in typical relaxor ferroelectrics^44^,^45^ Since the proposed microscopic origin of the Fano resonance in Li-doped KTN single crystal is the coupling between CP and TO_2_ mode, it is expected that the electric filed effect on CP and TO_2_ mode should be the similar fashion. From the Fig. 8, it is apparent that the intensity of CP and TO_2_ mode exhibits the similar dependences on the electric field. These results strongly support the coupling between the CP and the TO_2_ mode.

## Discussion

In the present study, the attention has been paid to clarify the microscopic origin of the Fano resonance in Li-doped KTN single crystals. The physical origin of the Fano resonance is discussed on the basis of different types of models and finally one model is chosen which reproduces the observed results appropriately. The Fano resonances reported in different types of materials may result from several different physical origins^24^,^25^,^26^,^27^,^28^,^29^,^30^. In BaTiO_3_ single crystals, the Fano resonance at around 175 cm^−1^ was attributed to the interference effect arising from the coupling of a single phonon state to a two acoustical phonon state through the anharmonic terms in the potential function^24^. In that case, the variation of the intensity of the acoustical mode and the Fano resonance should be the same in the wide temperature range. However, in Li-doped KTN crystals, the intensity of the 2TA mode did not exhibit any appreciable change in the cubic phase (Fig. 3c), whereas the intensity of the Fano resonance showed the noticeable temperature dependence (Fig. 4a,b). Therefore, the coupling between the TO_2_ phonon and the two acoustic phonons cannot be the origin of the Fano resonance in Li-doped KTN crystals.

Pinczuk *et al.* also suggested that the interference in BaTiO_3_ crystals at about 175 cm^−1^ can be due to the anharmonic coupling of the lowest frequency TO phonon with the higher frequency TO phonon via acoustic phonons^25^. In Li-doped KTN crystals, the lowest frequency TO phonon vanished at the *T*_C-T_, whereas the Fano resonance still existed above the *T*_C-T_ (Fig. 3c), and this fact again rules out the coupling of the lowest frequency TO phonon with the higher frequency TO phonon via acoustical phonons as the origin of our observation.

In the relaxor Ba(Zr_1/2_Ti_1/2_)O_3_ (BZT), the Fano resonance at around 155 cm^−1^ was caused by the interaction between the Ti and the Zr sublattices^28^. Interestingly, the Fano resonance in BZT persists up to 627 °C, which is far above the *T*_B_ ~ 177 °C at which dynamic PNRs start to appear. This is contrary to what was observed in the present observation, because the Fano resonance in Li-doped KTN crystals disappeared slightly above the T* at which the dynamic to static transition of PNRs begins.

The inelastic light scattering study reported that the rapid polarization fluctuations with the frequency up to the Fano resonance frequency within micro and nanoscopic polar regions interfere with a polar TO phonon resulting in the Fano resonance in non-relaxor SrTiO_3_ thin films^26^, and SrTiO_3_ and Ca_*x*_Sr_1−*x*_TiO_3_ nanocubes^27^, respectively. However, the situation in Li-doped KTN is perfectly different from the situation in refs. 26 and 27, where the micro and nanoscopic polar regions were arisen from incorporated impurities, most likely due to the oxygen vacancies. In contrast, the PNRs can be induced in KTN owing to the off-center displacements Nb ions at the B-site. In KTN and Li-doped KTN, the local polarization fluctuations of PNRs itself are much slower than that of the TO_2_ phonon frequency, and appear as the CP in a GHz frequency range^10^,^11^,^21^. Furthermore, it is significant that the value of the *q* gradually increased towards the *T*_C-T_ and became the maximum in the ferroelectric phase, where the local polarization fluctuations were totally absent. Therefore, the interference between the rapid polarization fluctuations within micro polar regions and the TO_2_ phonon in non-relaxor SrTiO_3_ films cannot be the origin of the Fano resonance in Li-doped KTN crystals.

According to the theory, the *q* parameter is inversely proportional to the density of continuum states (*ρ*) and the interaction strength (*V*)^46^,^47^. The *Г*_TO2_ of the Fano resonance is proportional to the *ρV*^*2*^. It is interesting to note that both *Г*_TO2_ and *q*^−1^ increased approximately linearly with the temperature towards the *T*_C-T_ in the ferroelectric phase as shown in Fig. 4d. These results reflect that the *V* does not vary strongly with the temperature, and the temperature dependence of both *Г*_TO2_ and *q*^−1^ was caused by the increase of the density of continuum states towards the *T*_C-T_. These results are similar to those observed in a ferroelectric semiconductor Sn_2_P_2_Se_6_^29^.

Since the Fano resonance in Li-doped KTN crystals is affected by the precursor dynamics in the cubic phase, there can be the correlation between the Fano resonance and PNRs. It is well established that the dynamic PNRs in KTN and Li-doped KTN crystals are observed as the CP^10^,^11^,^13^,^21^. Therefore, to clarify the physical origin of the Fano resonance, it is worth to compare the variation of the intensity of the CP and the Fano resonance with the temperature, angular, and electric field dependences of Raman spectra. It is clearly seen that the CP and the Fano resonance intensities showed the similar dependences on temperature, angular, and electric field, which demonstrates that the Fano resonance is driven by the CP. On the basis of these results, it is concluded that the coupling between the CP and the TO_2_ phonon can be the origin of the Fano resonance in Li-doped KTN single crystals^30^.

In summary, the microscopic origin of the Fano resonance in Li-doped KTN single crystals was investigated by the temperature, angular, and electric field dependences of Raman scattering. The breaking of local symmetry in the cubic phase due to the existence of PNRs with *E*(*x*, *y*) symmetry was observed by the intense first order Raman scattering, in which the first order scattering is forbidden in the cubic *Pm-3m* symmetry. In the VH Raman spectra, the remarkable field dependence of the Fano resonance intensity in the cubic phase can be the dynamical response of local polarizations to the electric field. The CP and TO_2_ phonon intensities showed the similar dependences on temperature, angular, and electric field. From these experimental results, it is concluded that the origin of the Fano resonance in Li-doped KTN crystals is the coupling between the polarization fluctuations of PNRs and the TO_2_ phonon, both belong to the *E*(*x*, *y*) symmetry.

## Additional Information
